# Cervical myelopathy symptom severity, posterior-based cervical surgical approach, and lower body mass index are associated with postoperative delirium: A retrospective observational study

**DOI:** 10.1016/j.xnsj.2025.100789

**Published:** 2025-09-11

**Authors:** Catherine R. Olinger, Pei-fu Chen, Sarah J. Lee, Daniel F. Waldschmidt, Reagan A. Grieser-Yoder, Lauren G. Havertape, Debra J. O’Connell-Moore, Lanchi B. Nguyen, Jill D. Corlette, Bradley J. Hindman, Matthew A. Howard

**Affiliations:** a Department of Orthopedics and Rehabilitation, University of IA Roy J. and Lucille A. Carver College of Medicine, 701 W Forevergreen Drive, North Liberty, IA, 52317, United States; b Department of Anesthesiology, Far Eastern Memorial Hospital, New Taipei City, Taiwan; c Department of Neurosurgery, University of IA Roy J. and Lucille A. Carver College of Medicine, 200 Hawkins Drive, IA City, Iowa, IA 52242, United States; d Institute for Clinical and Translational Science, University of IA, 200 Hawkins Drive, IA City, Iowa, IA 52242, United States; e Department of Anesthesia, University of IA Roy J. and Lucille A. Carver College of Medicine, 200 Hawkins Drive, IA City, Iowa, IA 52242, United States

**Keywords:** Cervical spine surgery, Cervical spondylotic myelopathy, Cervical radiculopathy, Delirium, Modified Japanese Orthopedic association scale, Surgical approach

## Abstract

**Background:**

Cervical spine surgery is often performed to alleviate symptoms of cervical spondylotic myelopathy (CSM) and/or cervical radiculopathy (CR). Although postoperative delirium (POD) is common after cervical spine surgery, it is not known if CSM, CSM symptom severity, and/or surgical approach (anterior vs. posterior) affect POD incidence or severity. The purpose of this study was to determine 1) If the preoperative diagnosis of CSM was an independent risk factor for POD incidence or severity; 2) Among patients who had CSM, which patient and intraoperative characteristics, including CSM symptom severity, were independently associated with POD incidence or severity.

**Methods:**

A retrospective search of the electronic medical record of a tertiary academic medical center identified patients undergoing cervical spine surgery. Patients who had: 1) POD assessments within the first 7 days of surgery (Delirium Observation Screening Scale [DOSS]; and 2) preoperative clinical diagnoses of CSM or CR were selected for analysis. Patient and surgical characteristics were extracted from the medical record, including CSM symptom severity (modified Japanese Orthopedic Association [mJOA] scores). Characteristics that were univariately associated with POD were included in multivariable models to determine characteristics that were independently associated with POD incidence and severity.

**Results:**

In the entire cohort (755 patients), POD incidence was (139/755) 18.4%, and 4 characteristics were independently associated with greater POD incidence: posterior-based surgical approach (adjusted odds ratio [aOR]=2.27, p=.0005), greater American Society of Anesthesiologists (ASA) class (aOR=1.66, p=.0432), obstructive sleep apnea (OSA) (aOR=1.76, p=.0280), and depression (aOR = 2.20, p=.0138). In this cohort, POD severity was independently associated with posterior-based surgical approach (Beta coefficient=0.4346, p=.0000) greater ASA class (Beta coefficient=0.1648, p=.0326), and lower preoperative hemoglobin (Beta coefficient=-0.0663, p=.0014). In the CSM subgroup (*n* = 629), POD severity was independently associated with posterior-based surgical approach (Beta coefficient=0.5527, p=.0002), OSA (Beta coefficient=0.4650, p=.0100), lower body mass index (BMI) (Beta coefficient = -0.0246, p=.0194) and lower (more severe) mJOA scores (Beta coefficient = -0.0465, p=.0197).

**Conclusions:**

For patients who have CSM, more severe symptoms (lower mJOA scores) and lower BMI were independently associated with greater POD severity. In addition, posterior-based surgical procedures were independently associated with greater POD incidence and severity.

## Introduction

Postoperative delirium (POD) occurs in 15–40% of patients, varying with patient and surgical characteristics [[1], [2], [3]]. POD is associated with a greater incidence of predischarge adverse events [1,4], greater length of stay [1,4], greater incidence of nonhome discharge, [1,4] greater readmissions, [1,4], delayed recovery of ambulation, [5] greater short- and long-term costs, [6] greater mortality, [1] and long-term cognitive decline. [7,8].

A recent meta-analysis of risk factors for POD after spine surgery reported cervical spine procedures were associated with a greater risk of POD *vs.* other spine procedures: 11 studies: odds ratio (OR)=1.71 (95% [Confidence Interval] CI 1.26–2.33), p=.001 [9]. The reason why cervical spine surgery may present a greater risk of POD compared with surgery at other spinal levels is unclear.

Cervical spine surgery is heterogeneous in terms of presenting pathologies and surgical approaches. If or how these many differences might affect POD after cervical spine surgery is unknown. In a retrospective study, Kim *et al*. reported patients who underwent cervical laminoplasty had a greater incidence of POD (19/78=24.3%) than patients who underwent ACDF (5/70=7.1%); OR=4.18 (95% CI 1.47–11.9), p=.005 [10]. However, these 2 groups differed in several key characteristics that were not included as variables in their multivariable model, the most important being: 1) the primary clinical indication for surgery (CSM *vs.* CR); and 2) the severity of preoperative CSM symptoms. We hypothesized that, rather than the surgical approach per se (*ie*, posterior *vs.* anterior), the presence and/or severity of preoperative CSM could have contributed to greater POD incidence in the laminoplasty group. In support of this hypothesis, a retrospective study of patients who underwent laminoplasty for CSM reported the preoperative Japanese Orthopaedic Association (JOA) scores of 10 patients who had POD (mean ± standard deviation:10.9±1.1) were numerically but not significantly less (worse) than JOA scores of 57 patients without POD (11.9±2.1), p=.13 [11]. This observation suggests CSM severity might be significantly associated with POD and might be detected with a larger cohort and/or with a greater range of JOA scores.

This retrospective observational study in patients who had undergone cervical spine surgery aimed to determine whether the preoperative clinical diagnosis (CSM vs. CR), surgical approach (anterior vs. posterior) or, in patients with CSM, the severity of CSM symptoms, were independent risk factors for POD.

## Methods

This study was approved by the IRB (number 202302241; approved April 6, 2023). The study performed at a single institution, extracting data from the electronic medical record (EMR) from 2012 to 2023 that included assessments of POD following cervical spine operations performed by both orthopedic and neurosurgery spine surgeons (Supplementary Table S1 Procedure Codes). For patients who had undergone more than 1 cervical spine operation, we included only the first operation. This process identified 3,081 cervical spine surgery patients.

**Fig. 1:**
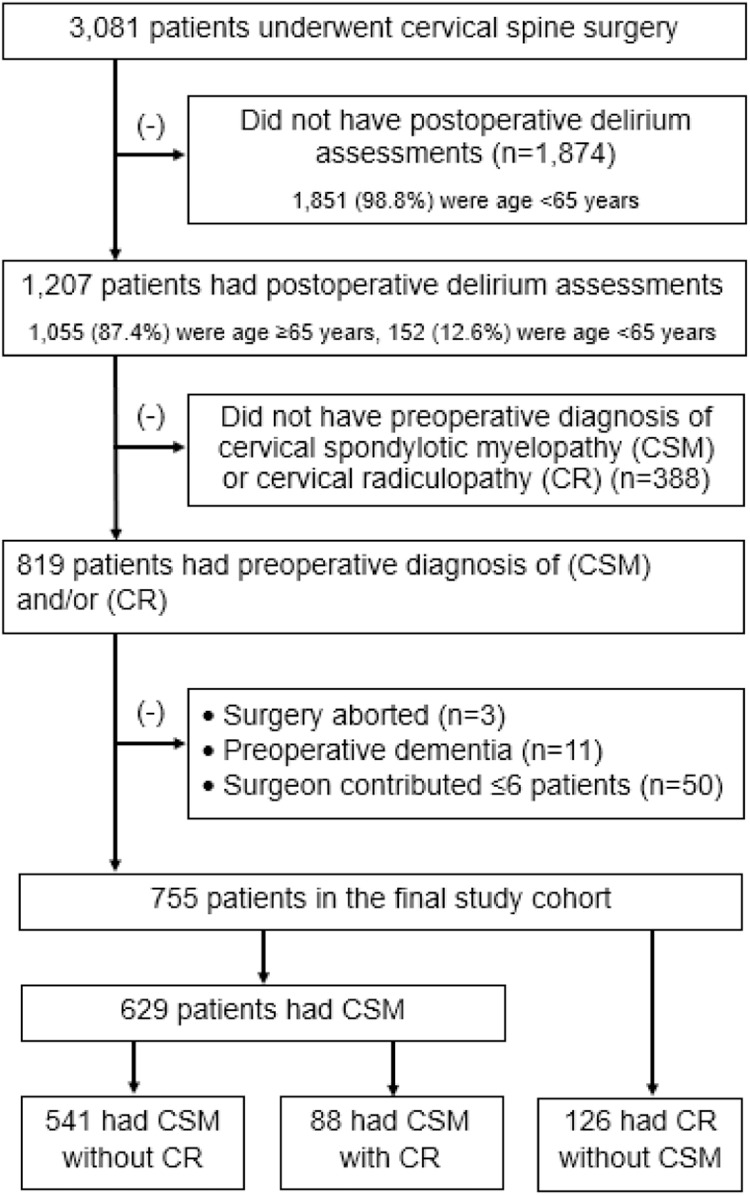
Flowchart of the study population.

In patients aged ≥ 65 years, twice daily delirium assessments were to be routinely made by ward nursing staff and entered into the EMR. Among the 1,078 patients aged ≥65 years, DOSS scores were recorded in 1,055 (97.9%). Nursing staff had the option to enter delirium assessments of patients younger than age 65. Delirium was assessed using the Delirium Observation Screening Scale (DOSS) which consists of 13 items; a score ≥3 was the delirium threshold [12]. Patients selected for this study had to have at least 1 DOSS score within 7 days of surgery. Ultimately, among patients who had postoperative DOSS scores (*n*=1,207), 452 patients were excluded (Fig. 1) which resulted in a final cohort of 755 cervical spine surgery patients who were classified into one of 3 preoperative clinical symptom subgroups: 1) CSM without cervical radiculopathy (CR) (*n*=541); 2) CSM with coexisting CR (*n*=88); and 3) CR without CSM (*n*=126) (Table 1 and Supplementary Table S2 Diagnosis Text). The first aim of this study was to determine if the preoperative clinical diagnosis of CSM was independently associated with delirium in patients undergoing cervical spine surgery. To do so required a subgroup of patients who did not have CSM. Thus, we included the CR without CSM subgroup (*n*=126) in the overall analysis of the entire cohort (*n*=755), serving as the reference group. Thereafter, to determine if CSM symptom severity was associated with delirium, we limited analysis to patients who had CSM (*n*=629).

**Table 1:** Characteristics of the entire cervical spine surgery cohort and subgroups.

Characteristic	Entire cohort (*n*=755)	CSM (*n*=629)	CSM subgroups		CR without CSM (*n*=126)*
			CSM without CR (*n*=541)	CSM with CR (*n*=88)*	
Patient					
Age (years)	70 (66-75)	71 [67, 76]	71 [67, 76]	68 [65, 72]	66 [55, 71]
Gender: Male	414 (54.8)	354 (56.3)	316 (58.4)	38 (43.2)	60 (47.6)
Body mass index (kg/m^2^)†	29.38 [25.6, 33.4]	29.2 [25.6, 33.2]	29.2 [25.6, 33.1]	29.3 [26.1, 33.6]	30.7 [25.7, 35.9]
Race: White	712 (94.3)	594 (94.4)	510 (94.3)	84 (95.5)	118 (93.7)
Ethnicity: Non-Hispanic	745 (98.7)	620 (98.6)	535 (98.9)	85 (96.6)	125 (99.2)
ASA Physical Class					
1	8 (1.1)	5 (0.8)	2 (0.4)	3 (3.4)	3 (2.4)
2	222 (29.4)	168 (26.7)	135 (25.0)	33 (37.5)	54 (42.9)
3	495 (65.6)	429 (68.2)	381 (70.4)	48 (54.5)	66 (52.4)
4	30 (4.0)	27 (4.3)	23 (4.3)	4 (4.5)	3 (2.4)
Current smoking	108 (14.3)	85 (13.5)	70 (12.9)	15 (17.0)	23 (18.3)
Alcohol use	278 (36.8)	227 (36.1)	192 (35.5)	35 (39.8)	51 (40.5)
Diabetes mellitus	154 (20.4)	135 (21.5)	115 (21.3)	20 (22.7)	19 (15.1)
Hypertension	342 (45.3)	291 (46.3)	248 (45.8)	43 (48.9)	51 (40.5)
Hypothyroidism	71 (9.4%)	61 (9.7)	50 (9.2)	11 (12.5)	10 (7.9)
Depression	68 (9.0%)	52 (8.3)	39 (7.2)	13 (14.8)	16 (12.7)
Obstructive sleep apnea	119 (15.8)	98 (15.6)	82 (15.2)	16 (18.2)	21 (16.7)
Preoperative Hemoglobin (g/dL)	12.9 [11.5, 14.0]	12.7 [11.3, 14.0]	12.7 [11.2, 13.9]	13.7 [12.4, 14.4]	13.3 [12.2, 14.3]
Preoperative Creatinine (mg/dL)	0.9 [0.7, 1.1]	0.9 [0.7, 1.1]	0.9 [0.7, 1.1]	0.9 [0.7, 1.0]	0.9 [0.7, 1.0]
mJOA score (points)‡	12.0 [9.0, 14.0]	11.5 [9.0, 14.0]	11.0 [9.0, 14.0]	12.0 [10.0, 13.3]	13.0 [11.0, 15.0]
Surgical					
Posterior-based surgical approach	331 (43.8)	302 (48.0)	283 (52.3)	19 (21.6)	29 (23.0)
Procedure duration (min)	169 [119, 230]	178 [121, 235]	185 [127, 240]	131 [98, 186]	134 [104, 180]
Estimated blood loss (mL)	80 [25, 200]	100 [30, 200]	100 [40, 200]	50 [19, 150]	50 [20, 100]
Surgeon					
A	161 (21.3)	147 (23.4)	138 (25.5)	9 (10.2)	14 (11.1)
B	113 (15.0)	87 (13.8)	53 (9.8)	34 (38.6)	26 (20.6)
C	104 (13.8)	80 (12.7)	70 (12.9)	10 (11.4)	24 (19.0)
D	83 (11.0)	58 (9.2)	45 (8.3)	13 (14.8)	25 (19.8)
E	71 (9.4)	63 (10.0)	60 (11.1)	3 (3.4)	8 (6.3)
F	77 (10.2)	66 (10.5)	63 (11.6)	3 (3.4)	11 (8.7)
G	45 (6.0%)	40 (6.4)	36 (6.7)	4 (4.5)	5 (4.0)
H	25 (3.3%)	21 (3.3)	17 (3.1)	4 (4.5)	4 (3.2)
I	19 (2.5%)	19 (3.0)	18 (3.3)	1 (1.1)	0 (0.0)
J	17 (2.3%)	11 (1.7)	9 (1.7)	2 (2.3)	6 (4.8)
K	17 (2.3%)	14 (2.2)	10 (1.8)	4 (4.5)	3 (2.4)
L	12 (1.6%)	12 (1.9)	12 (2.2)	0 (0.0)	0 (0.0)
M	11 (1.5%)	11 (1.7)	10 (1.8)	1 (1.1)	0 (0.0)
Postoperative					
Delirium incidence	139 (18.4)	126 (20.0)	115 (21.3)	11 (12.5)	13 (10.3)
Maximum delirium severity (points)	0 [0, 1]	0 [0, 2]	0 [0, 2]	0 [0, 0]	0 [0, 0]
Length of stay (days)	3 [1, 6]	4 [2, 7]	4 [2, 7]	2 [1, 4]	1 [1,3]
Nonhome discharge	304 (40.3)	287 (45.6)	266 (49.2)	21 (23.9)	17 (13.5)

All data expressed as median [25th, 75th percentile] for continuous variables or n (%) for categorical variables. p-values ≤.05 are in bold font. Abbreviations: ASA, American Society of Anesthesiologists; CR, Cervical radiculopathy; CSM, Cervical spondylotic myelopathy; mJOA, modified Japanese Orthopaedic Association; SMD, standardized mean difference.

⁎ The CSM with CR subgroup (n=88) and the CR without CR subgroup (n=126) did not significantly differ in any characteristic except mJOA score (P=0.0183, SMD = 0.5065).

† Preoperative body mass index values were present in 404/541 (74.7%) of patients with CSM without CR, 70/88 (79.5%) of patients with CSM with CR, and 100/126 (79.4%) of patients with CR without CSM.

‡ Preoperative mJOA scores were present in 308/541 (56.9%) of patients with CSM without CR, 44/88 (50.0%) of patients with CSM with CR, and 65/126 (51.6%) patients with CR without CSM.

We recorded multiple characteristics: age, gender, body mass index (BMI), race, ethnicity, American Society of Anesthesiologists (ASA) physical status class, current and former tobacco use, and alcohol use were recorded. Further, we assessed 6 major preoperative comorbidities (diabetes mellitus [DM], hypertension, hypothyroidism, depression, obstructive sleep apnea [OSA], and dementia) (Supplementary Table S3 Comorbidity Text). Preoperative physiologic measures included preoperative hemoglobin and creatinine concentrations and mJOA scores [13], which quantified preoperative CSM symptom severity.

We also assessed multiple surgical variables focusing most critically on anterior (*n*=424) or posterior based surgery (*n*=331), with the latter including combined anterior and posterior procedures (*n*=18). The attending surgeon of record was extracted from the intraoperative case log. To preserve confidentiality, each surgeon was recoded to an anonymized identifier (Surgeons A–M). In univariate analyses, Surgeon was entered as a k-level factor with odds ratios for each level relative to the reference surgeon and a global likelihood-ratio p value for the overall surgeon effect. For multivariable models, we applied a prespecified rule: if the global LR test was significant, we retained the full multilevel surgeon factor; if not, we included only those surgeon indicators with univariate p<.05 to avoid over-parameterization. We recorded POD incidence/severity, length of stay, and discharge disposition (home vs. nonhome). Discharge disposition (“home” vs “nonhome”) was based on the destination recorded at discharge; preoperative discharge plans were not consistently documented and could not be analyzed.

All data were obtained from original records without imputation of missing values. Continuous variables were summarized using medians and interquartile ranges, with comparisons between groups made using Wilcoxon rank sum tests, while categorical variables were summarized using counts and percentages, with comparisons conducted via Pearson’s chi-square test. Standardized mean differences (SMD) [14] were calculated, with values ≥0.200 indicating noncomparability between groups [15,16]. POD incidence was defined as any DOSS score ≥3 before discharge or postoperative day 7, whichever came first, while POD severity was determined by the highest DOSS score within this period. Univariate analyses were performed to assess associations between patient and surgical characteristics with POD incidence (logistic regression) and POD severity (robust regression).

Characteristics with p<.05 in univariate analyses were included in multivariable logistic regression for POD incidence and multivariable robust regression with M-estimation and Huber weighting [17] for POD severity. Odds ratios, beta coefficients, and p values were reported as appropriate, with p<.0001 denoted as <0.0001. Multicollinearity was assessed using variance inflation factors (VIF), with values <2.5 indicating low collinearity [18]. The generalized VIF (GVIF) was adjusted for degrees of freedom to ensure consistency across categorical predictors [19]. All p values were 2-sided, with significance set at <0.05 without adjustment for multiple comparisons. Analyses were conducted using R version 4.1.3, utilizing relevant statistical packages including “tableone,” “stats,” “MASS,” “sfsmisc,” “lmtest,” “DescTools,” and “car.”

## Results

**Fig. 2:**
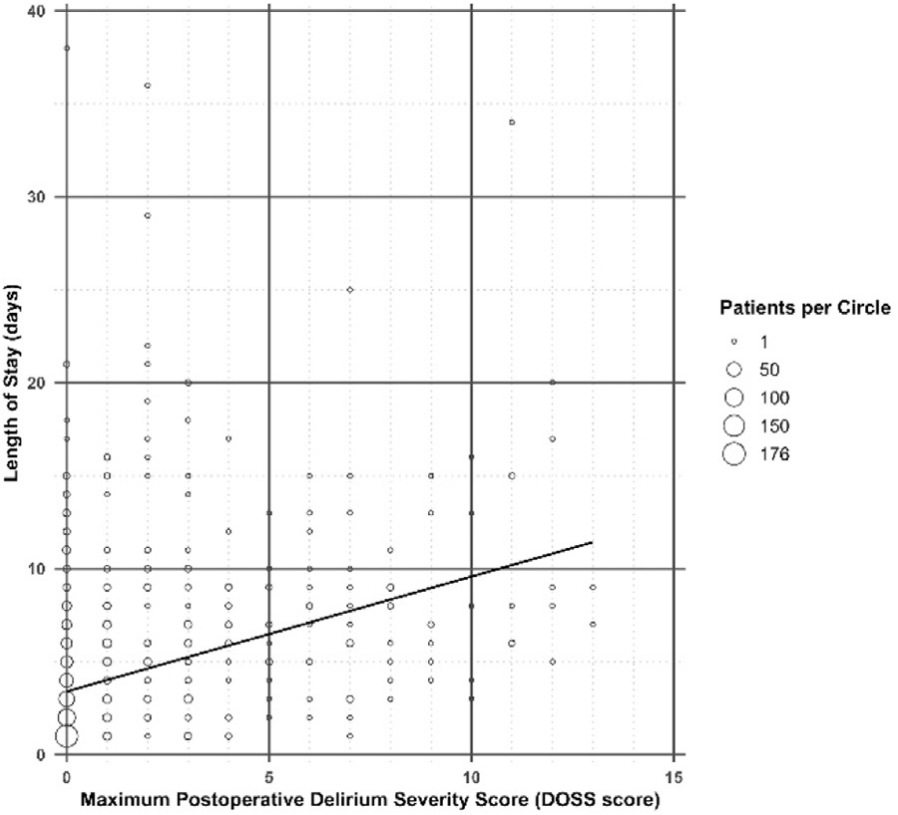
Postoperative length of stay *vs.* maximum postoperative delirium severity (*n*=755). The robust regression line is shown, Beta coefficient=0.6173 (SE=0.0440), p<.0001.

POD occurred in 18.4% of patients (139/755) and was associated with a longer hospital stay (median 7 vs. 3 days, p<.0001) and a higher rate of nonhome discharge (70.5% vs. 33.4%, p<.0001) (Supplementary Table S4 Delirium Characteristics). POD severity, ranging from 1 to 13, correlated with both length of stay (Beta coefficient=0.6173 [SE=0.0440], p<.000; Fig. 2) and increased likelihood of nonhome discharge (OR=1.39 [95% CI 1.29–1.51], p<.0001). Among the entire cohort (*n*=755), patients were categorized into 3 subgroups: CSM without CR (*n*=541), CSM with CR (*n*=88), and CR without CSM (*n*=126) (Table 1). The incidence and severity of POD varied across these subgroups (Supplementary Table S5 Characteristics Among Groups).

Within the entire cohort (*n* = 755), univariate analysis identified 9 characteristics associated with increased POD incidence: posterior-based surgical approach, higher ASA class, OSA, depression, diabetes, older age, longer procedure duration, CSM without CR, and surgeon (Table 2). A higher preoperative hemoglobin concentration was associated with a lower risk of POD (OR <1.00). Multivariable analysis showed that posterior-based surgical approach, higher ASA class, OSA, and depression were independent risk factors, while the diagnosis of CSM (with [aOR=1.22, p=.6709] or without CR [aOR=1.34, p=.4006]) was not. Similarly, greater POD severity was univariately associated with posterior-based surgery, higher ASA class, older age, OSA, diabetes, longer procedure duration, estimated blood loss, CSM without CR, and surgeon, while higher preoperative hemoglobin was associated with lower POD severity (Table 3). Multivariable analysis showed that posterior-based surgical approach, higher ASA class, and lower preoperative hemoglobin were independently associated with greater POD severity.

**Table 2:** Univariate and multivariable analysis of characteristics associated with delirium incidence in the entire cervical spine surgery cohort (*n*=755)

Characteristic	Univariate (*n*=755)			Multivariable logistic regression model (c-statistic=0.73)			
	OR	95% CI	p-value	aOR	95% CI	p-value	GVIF
Posterior-based surgical approach	3.34	2.27-4.97	**<.0001**	2.27	1.43-3.65	**.0005**	1.16
ASA class (3 or 4 vs 1 or 2)	2.15	1.38-3.46	**.0010**	1.66	1.03-2.77	**.0432**	1.03
Obstructive Sleep Apnea	1.92	1.21-2.99	**.0047**	1.76	1.05-2.90	**.0280**	1.06
Depression	1.83	1.02-3.18	**.0357**	2.20	1.16-4.10	**.0138**	1.04
Diabetes mellitus	1.70	1.11-2.58	**.0138**	1.26	0.78-2.00	.3433	1.06
Age (years)	1.04	1.02-1.07	**.0012**	1.03	1.00-1.06	.0533	1.10
Procedure duration (min)	1.00	1.00-1.01	**.0006**	1.00	1.00-1.00	.2826	1.17
Preoperative hemoglobin concentration (gm/dL)	0.84	0.76-0.93	**.0008**	0.97	0.86-1.08	.5387	1.08
Clinical diagnosis			**0034**			.6572	1.05
CR without CSM (reference, *n*=126)							
CSM without CR (*n*=541)	2.35	1.32-4.51	**.0061**	1.34	0.70-2.72	.4006	
CSM with CR (*n*=88)	1.24	0.52-2.92	.6190	1.22	0.48-3.04	.6709	
Surgeon			**.0045**			.4233*	1.02
A (reference)							
B	0.34	0.15-0.69	**.0044**	0.56	0.24-1.23	.1626	
C	0.73	0.38-1.35	.3199	0.77	0.39-1.49	.4406	
D	0.27	0.10-0.63	**.0049**	0.50	0.17-1.27	.1709	
E	1.09	0.56-2.09	.7912	0.90	0.44-1.81	.7789	
F	1.22	0.64-2.27	.5390	1.28	0.64-2.51	.4848	
G	1.26	0.58-2.65	.5463	1.33	0.58-2.92	.4890	
H	0.66	0.18-1.87	.4740	0.59	0.16-1.78	.3830	
I	1.60	0.53-4.37	.3723	1.31	0.42-3.72	.6218	
J	1.07	0.29-3.23	.9126	1.25	0.31-4.30	.7372	
K	0.46	0.07-1.74	.3211	0.76	0.11-3.13	.7392	
L	0.32	0.02-1.71	.2773	0.17	0.01-0.94	.0953	
M	1.30	0.27-4.77	.7073	0.79	0.16-3.11	.7546	

P values less than or equal to 0.05 are in **bold** font.

Abbreviations: aOR, adjusted odds ratio; ASA, American Society of Anesthesiologists; CI, confidence interval, CR, cervical myelopathy, CSM, cervical spondylotic myelopathy; GVIF, generalized variance inflation factor; OR, odds ratio. p values ≤.05 are in bold font.

⁎ p-values for the categorical Surgeon variable (levels A to M) were calculated using a likelihood ratio test comparing the full model (including Surgeon) to the null model (excluding Surgeon).

**Table 3:** Univariate and multivariable analysis of characteristics associated with delirium severity in the entire cervical spine surgery cohort (*n*=755)

Characteristic	Univariate (*n*=755)			Multivariable robust regression model (c-statistic=0.69)			
	Beta Coefficient	SE	p-value	Beta coefficient	SE	p-value	GVIF
Posterior-based surgical approach	0.6130	0.0755	**<.0001**	0.4346	0.0860	**<.0001**	1.26
ASA class (3 or 4 vs 1 or 2)	0.2886	0.0735	**.0001**	0.1648	0.0787	**.0326**	1.06
Age (years)	0.0120	0.0031	**.0001**	0.0054	0.0041	.1771	1.13
Obstructive sleep apnea	0.0018	0.0007	**.0223**	0.1557	0.0976	.1232	1.05
Diabetes mellitus	0.0013	0.0004	**.0066**	0.0633	0.0901	.4982	1.07
Procedure duration (min)	0.0012	0.0003	**.0001**	0.0003	0.0005	.4896	1.27
Estimated blood loss (mL)	0.0003	0.0001	**.0003**	−0.0001	0.0001	.2013	1.23
Preoperative hemoglobin concentration (mg/dL)	−0.1038	0.0164	**<.0001**	−0.0663	0.0205	**.0014**	1.11
Clinical diagnosis		**0.0009**				.5585	1.08
CR without CSM (reference, *n*=126)							
CSM without CR (*n*=541)	0.2827	0.0870	**.0009**	0.0469	0.1020	.6398	
CSM with CR (*n*=88)	0.0524	0.1222	.6578	0.0504	0.1321	.6954	
Surgeon			**.0004**			.9977*	1.03
A (reference)							
B	−0.2988	0.1076	**.0050**	−0.0649	0.1256	.6023	
C	−0.0255	0.1103	.8178	0.0250	0.1198	.8352	
D	−0.3493	0.1185	**.0025**	−0.0564	0.1377	.6762	
E	−0.0705	0.1249	.5796	−0.1867	0.1360	.1793	
F	0.1918	0.1215	.1261	0.1631	0.1329	.2319	
G	0.0234	0.1478	.8775	0.0873	0.1586	.5931	
H	−0.0207	0.1885	.9136	0.0287	0.2157	.8942	
I	0.0987	0.2127	.6552	0.0717	0.2280	.7613	
J	−0.0192	0.2236	.9334	0.0752	0.2437	.7628	
K	−0.3159	0.2236	.1507	−0.1248	0.2431	.5974	
L	−0.1309	0.2624	.6081	−0.5179	0.2839	.0632	
M	0.7528	0.2732	**.0101**	0.4361	0.2948	.1594	

P values less than or equal to 0.05 are in **bold** font.

Abbreviations: ASA, American Society of Anesthesiologists; CR, cervical myelopathy; CSM, cervical spondylotic myelopathy; GVIF, generalized variance inflation factor; SE, standard error. p values ≤.05 are in bold font.

⁎ p-values for the categorical Surgeon variable (levels A to M) were calculated using a likelihood ratio test comparing the full model (including Surgeon) to the null model (excluding Surgeon).

Within the CSM subgroup (*n*=629), 56.0% had preoperative mJOA scores, with no significant difference in score availability between the 2 CSM subgroups. Six characteristics differed between patients with and without mJOA scores, including age, ASA class, smoking status, OSA, preoperative hemoglobin, and surgeon (Supplementary Table S6 mJOA Characteristics). However, mJOA scores were not significantly associated with POD incidence (OR=0.94, p=.1329) and were therefore excluded from multivariable modeling. Independent risk factors for POD incidence in the CSM cohort were: posterior-based surgical approach and OSA (Table 4) Greater POD severity was univariately associated with posterior-based surgery, ASA class, OSA, older age, longer procedure duration, and greater estimated blood loss, while higher BMI, less severe mJOA scores, greater preoperative hemoglobin, and a diagnosis of CR were linked to lower POD severity (Table 5). In the multivariable logistic regression model of POD severity (Table 5), posterior-based surgical approach, OSA, lower BMI, and lower (worse) mJOA scores were independently associated with greater POD severity.

**Table 4:** Univariate and multivariable analysis of characteristics associated with delirium incidence in the cervical spondylotic myelopathy (CSM) cohort (n=629)

Characteristic	Univariate (*n*=629)			Multivariable Logistic Regression Model (c-statistic = 0.70)			
	OR	95% CI	p-value	aOR	95% CI	p-value	GVIF
Posterior-based surgical approach	3.27	2.16-5.04	**<.0001**	2.40	1.49-3.94	**.0004**	1.28
Obstructive sleep apnea	2.00	1.22-3.22	**.0050**	1.99	1.18-3.30	**.0085**	1.04
ASA class (3 or 4 vs 1 or 2)	1.79	1.12-2.96	**.0185**	1.54	0.94-2.59	.0944	1.02
Age (years)	1.03	1.00-1.06	**.0251**	1.02	0.99-1.05	.2065	1.08
Procedure duration (min)	1.00	1.00-1.00	**.0046**	1.00	1.00-1.00	.5890	1.19
Preoperative hemoglobin concentration (gm/dL)	0.86	0.78-0.96	**.0047**	0.95	0.84-1.06	.3352	1.10
Surgeon (Surgeon *A* = reference)			.0524				
B*	0.34	0.14-0.73	**.0094**	0.55	0.23-1.19	.1531	1.11
D*	0.38	0.14-0.91	**.0429**	0.66	0.23-1.63	.4032	1.16

P values less than or equal to 0.05 are in **bold** font.

Abbreviations: aOR, adjusted odds ratio; CI, confidence interval, CR, cervical myelopathy; CSM, cervical spondylotic myelopathy; GVIF, generalized variance inflation factor; OR, odds ratio. p-values ≤.05 are in bold font.

⁎ Only Surgeons B and D, from the multicategorical variable representing surgeons, were significantly associated with the incidence of delirium; therefore, only these 2 categories were included in the multivariable model.

**Table 5:** Univariate and multivariable analysis of characteristics associated with delirium severity score in patients having cervical spondylotic myelopathy (CSM), (*n* = 629).

Characteristic	Univariate (*n*=629, except for BMI *n*=474 and mJOA *n*=352)			Multivariable Robust Regression Model (*n*=289, c-statistic = 0.67)			
	Beta Coefficient	SE	p-value	Beta Coefficient	SE	p-value	GVIF
Posterior-based surgical approach	0.6385	0.0863	**<.0001**	0.5527	0.1426	**.0002**	1.34
ASA class (3 or 4 vs 1 or 2)	0.2715	0.0959	**.0042**	0.0794	0.1457	.5824	1.20
Obstructive sleep apnea	0.1586	0.0768	**.0467**	0.4650	0.1696	**.0100**	1.15
Age (years)	0.0111	0.0046	**.0135**	−0.0049	0.0119	.6836	1.24
Procedure duration (min)	0.0014	0.0004	**.0008**	−0.0001	0.0008	.9463	1.50
Estimated blood loss (mL)	0.0002	0.0001	**.0274**	−0.0003	0.0002	.1050	1.37
Preoperative body mass index (kg/m^2^)	−0.0121	0.0061	**.0441**	−0.0246	0.0105	**.0194**	1.15
Preoperative mJOA score	−0.0425	0.0147	**.0045**	−0.0465	0.0195	**.0197**	1.21
Preoperative hemoglobin concentration (gm/dL)	−0.1171	0.0206	**<.0001**	−0.0420	0.0346	.2362	1.18
Clinical diagnosis of CR	−0.2303	0.1081	**.0282**	−0.0241	0.1934	.8977	1.05
Current smoking*	0.0239	0.0900	.7927	−0.0946	0.2295	.6793	1.22
Surgeon (Surgeon *A* = reference)			.0706				
B†	−0.3520	0.1527	**.0195**	−0.0203	0.2534	.9348	1.09
D†	−0.4023	0.1750	**.0191**	−0.0957	0.2322	.6690	1.17

P values less than or equal to 0.05 are in **bold** font.

Abbreviations: ASA, American Society of Anesthesiologists; CI, confidence interval, CR, cervical myelopathy, CSM, cervical spondylotic myelopathy; GVIF, generalized variance inflation factor; mJOA, modified Japanese Orthopedic Association; SE, standard error. p values ≤.05 are in bold font.

⁎ The occurrence of current smoking differed significantly between patients with mJOA and those without (Supplemental Table S6), leading to its inclusion in the multivariable model.

† Only Surgeons B and D, from the multicategorical variable representing surgeons, were significantly associated with the severity of delirium; therefore, only these 2 categories were included in the multivariable model.

Among patients in the CSM without CR subgroup (*n*=541), posterior- and anterior-based surgical approaches were used nearly equally (52.3% vs. 47.7%, respectively) (Table 1). Patients undergoing posterior procedures were older, had lower preoperative hemoglobin, longer surgical duration, greater intraoperative blood loss, and were treated by different surgeons (Supplementary Table S7 Surgical Approach Characteristics). Even after adjusting for these factors, posterior-based surgery remained a significant independent predictor of increased POD incidence (OR=3.46, [95% CI = 2.01–6.07], p<.0001) and greater POD severity (Beta coefficient= 0.7149, p<.0001) (Supplementary Table S8 Approach POD Incidence Model and Supplementary Table S9 Approach POD Severity Model).

## Discussion

This retrospective study found that a preoperative diagnosis of cervical spondylotic myelopathy (CSM) was not independently associated with postoperative delirium (POD) incidence or severity, regardless of the presence of concomitant cervical radiculopathy (CR) (Table 2, Table 3). However, among patients with CSM, lower preoperative mJOA scores—indicating greater disease severity—were independently associated with increased POD severity (Table 5), but not POD incidence (Table 4), which is consistent with prior work by Kin et al. [16]. These findings suggest that mJOA scores may be a more sensitive predictor of POD severity than binary CSM diagnostic classifications. This may be because greater CSM symptom severity indicates greater underlying alterations in cortical functional connectivity that may increase vulnerability to the disturbances causing postoperative delirium [[20], [21], [22], [23], [24], [25], [26], [27]].

Posterior-based surgical approaches were consistently associated with increased POD incidence and severity in both the entire and CSM-only cohorts, even after adjusting for surgeon, ASA class, and other characteristics. In the CSM without CR subgroup, posterior and anterior surgical approaches were used with similar frequency (Table 1), strengthening the case that the surgical approach, not clinical indication, drives this association. Posterior procedures in our cohort were associated with longer operative times and greater blood loss (Supplementary Table S7), supporting the hypothesis that greater intraoperative tissue trauma contributes to greater postoperative systemic and neuroinflammatory responses [[28], [29], [30], [31]], a known pathophysiologic mechanism for POD [[30], [31], [32]]. Prior studies linking POD to CRP levels [32] and number of instrumented levels [33] are consistent with our findings and support this mechanistic link.

Other independent predictors of POD included higher ASA class, obstructive sleep apnea (OSA), depression, lower preoperative hemoglobin, and lower BMI. Most of these factors have been previously reported in spine surgery cohorts [9,34,35]. Notably, lower BMI was independently associated with increased POD severity in CSM patients, a novel finding in spine surgery. This may be attributable to frailty or sarcopenia, conditions associated with altered inflammatory profiles [[36], [37], [38]] and cognitive vulnerability [41]. Additionally, low BMI has been linked to higher preoperative CSF tau concentrations, a potential POD biomarker [39].

### Limitations

Because this is a retrospective study, it is vulnerable to selection bias and misclassification bias. Retrospective studies should be considered as generating hypotheses rather than testing them. Accordingly, our findings need to be verified in future studies. Several limitations warrant specific consideration.

Although we observed significant associations between POD incidence and severity and both length of stay and nonhome discharge, the criteria upon which discharge decisions were based is unknown. Accordingly, we cannot be sure that delirium per se was a direct and independent determinant.

POD was assessed using the Delirium Observation Screening Scale (DOSS), a nursing-based tool that lacks structured cognitive evaluation. While it performs well for hyperactive delirium, it is less sensitive to hypoactive and mixed subtypes [40], likely leading to underestimation of true POD incidence. Additionally, mJOA scores were missing in 44% of patients with a CSM diagnosis. Although missingness was addressed in multivariable models, this may have reduced statistical power.

Diagnostic classifications of CSM and CR were based on medical record text searches and are subject to clinician variability and potential misclassification. Multivariable models adjusted for characteristics differing between diagnosed and undiagnosed patients, but residual confounding remains possible. Furthermore, surgical approach was not randomized, and surgeon practice patterns varied by subgroup (Table 1). However, surgeon was not an independent predictor in any model, suggesting the association between surgical approach and POD is robust.

Interpretation should also consider that multiple statistical tests were conducted without formal p-value adjustments for multiple comparisons to maintain exploratory power, although the primary finding regarding surgical approach withstood conservative correction.

Lastly, the study was conducted at a single academic center with limited racial and ethnic diversity, which may limit generalizability.

## Conclusions

In this study of older adults undergoing cervical spine surgery, greater CSM severity (as measured by lower mJOA scores) was independently associated with increased POD severity. Posterior-based surgical procedures were robustly associated with both POD incidence and severity, possibly reflecting greater intraoperative tissue trauma and inflammatory response. Lower BMI was also independently associated with increased POD severity in patients having CSM. These findings highlight the importance of myelopathic symptom severity, surgical approach, and frailty-related measures in determining POD risk and warrant validation in future prospective studies.

## Funding

Funding was provided by the University of IA Department of Neurosurgery and Department of Orthopedics and Rehabilitation. The Institute for Clinical and Translational Science is funded by the National Center For Advancing Translational Sciences of the 10.13039/100000002National Institutes of Health under Award Number UL1TR004403. This article is solely the responsibility of the authors and does not necessarily represent the official views of the National Institutes of Health.

## CRediT authorship contribution statement

**Catherine R. Olinger:** Conceptualization, Funding acquisition, Methodology, Project administration, Supervision, Writing – original draft, Writing – review & editing. **Pei-fu Chen:** Data curation, Formal analysis, Methodology, Software, Validation, Visualization, Writing – original draft, Writing – review & editing. **Sarah J. Lee:** Investigation, Writing – review & editing. **Daniel F. Waldschmidt:** Investigation, Writing – review & editing. **Reagan A. Grieser-Yoder:** Investigation, Writing – review & editing. **Lauren G. Havertape:** Investigation, Writing – review & editing. **Debra J. O’Connell-Moore:** Project administration, Supervision, Writing – review & editing. **Lanchi B. Nguyen:** Investigation, Writing – review & editing. **Jill D. Corlette:** Data curation, Software, Resources, Writing – review & editing. **Bradley J. Hindman:** Conceptualization, Data curation, Investigation, Methodology, Project administration, Supervision, Validation, Visualization, Writing – original draft, Writing – review & editing. **Matthew A. Howard:** Conceptualization, Funding acquisition, Project administration, Supervision, Writing – review & editing.

## Declaration of competing interest

One or more of the authors declare financial or professional relationships on ICMJE-TSJ disclosure form.

## Appendix Supplementary materials

Image, application 1

## References

[1] Watt J., Tricco A.C., Talbot-Hamon C. (2018). Identifying older adults at risk of delirium following elective surgery: a systematic review and meta- analysis. J Gen Intern Med.

[2] Wilson J.E., Mart M.F., Cunningham C. (2020). Delirium. Nat Rev Dis Prim.

[3] Deeken F., Sánchez A., Rapp M.A. (2022). Outcomes of a delirium prevention program in older persons after elective surgery: a stepped-wedge cluster randomized trial. JAMA Surg.

[4] Gleason L.J., Schmitt E.M., Kosar C.M. (2015). Effect of delirium and other major complications on outcomes after elective surgery in older adults. JAMA Surg.

[5] Robinson T.N., Kovar A., Carmichael H., Overbey D.M., Goode C., Jones T.S. (2021). Postoperative delirium is associated with decreased recovery of ambulation one-month after surgery. Am J Surg.

[6] Gou R.Y., Hshieh T.T., Marcantonio E.R. (2021). One-year Medicare costs associated with delirium in older patients undergoing major elective surgery. JAMA Surg.

[7] Huang H., Li H., Zhang X. (2021). Association of postoperative delirium with cognitive outcomes: a meta-analysis. J Clin Anesth.

[8] Kunicki Z.J., Ngo L.H., Marcantonio E.R. (2023). Six-year cognitive trajectory in older adults following major surgery and delirium. JAMA Intern Med.

[9] Luo M., Wang D., Shi Y. (2024). Risk factors for postoperative delirium following spine surgery: a meta-analysis of 50 cohort studies with 1.1. million participants. Heliyon.

[10] Kim N., Kim T.H., Oh J.K., Lim J., Lee K.U., Kim S.W. (2022). Analysis of the incidence and risk factors of postoperative delirium in patients with degenerative cervical myelopathy. Neurospine.

[11] Kin K., Yasuhara T., Tomita Y., Umakoshi M., Morimoto J., Date I. (2019). SF-35 scores predict postoperative delirium after surgery for cervical spondylotic myelopathy. J Neurosurg Spine.

[12] Gavinski K., Carnahan R., Weckmann M. (2016). Validation of the delirium observation screening scale (DOS) in a hospitalized older population. J Hosp Med.

[13] Kato S., Oshima Y., Oka H. (2015). Comparison of the Japanese Orthopaedic Association (JOA) score and modified JOA (mJOA) score for the assessment of cervical myelopathy: a multicenter observational study. PLoS One.

[14] Hedges L.V., Olkin I. (1985).

[15] Austin P.C. (2009). Balance diagnostics for comparing the distribution of baseline covariates between treatment groups in propensity-score matched samples. Stat Med.

[16] Austin P.C. (2011). An introduction to propensity score methods for reducing the effects of confounding in observational studies. Multivariate Behav Res.

[17] Huber P.J. (1981).

[18] Johnston R., Jones K., Manley D. (2018). Confounding and collinearity in regression analysis: a cautionary tale and an alternative procedure, illustrated by studies of British voting behaviour. Qual Quant.

[19] Fox J., Monett G. (1992). Generalized collinearity diagnostics. J Am Stat Assoc.

[20] Khan A.F., Muhammad F., Mohammadi E. (2024). Beyond the aging spine—a systematic review of functional changes in the human brain in cervical spondylotic myelopathy. Geroscience.

[21] Woodworth D.C., Holly L.T., Salamon N., Ellingson B.M. (2018). Resting-state functional magnetic resonance imaging connectivity of the brain is associated with altered sensorimotor function in patients with cervical spondylosis. World Neurosurg.

[22] Cao Y., Zhan Y., Du M. (2021). Disruption of human brain connectivity networks in patients with cervical spondylotic myelopathy. Quant Imag Med Surg.

[23] Eto F., Inomata K., Sakashita K. (2022). Postoperative changes in resting state functional connectivity and clinical scores in patients with cervical myelopathy. World Neurosurg.

[24] Shao Z., Tan Y., Zhan Y., He L. (2024). Modular organization of functional brain networks in patients with degenerative cervical myelopathy. Sci Rpt.

[25] Tan Y., Shao Z., Wu K., Zhou F., He L. (2024). Resting-state brain plasticity is associated with the severity in cervical spondylotic myelopathy. BMC Musculoskelet Disord.

[26] Zhao G., Zhan Y., Zha J., Cao Y., Zhou F., He L. (2023). Abnormal intrinsic brain functional network dynamics in patients with cervical spondylotic myelopathy. Cogn Neurodyn.

[27] Tanabe S., Mohanty R., Lindroth H. (2020). Cohort study into the neural correlates of postoperative delirium: the roles of connectivity and slow-wave activity. Br J Anaesth.

[28] Watt D.G., Horgan P.G., McMillan D.C. (2015). Routine clinical markers of the magnitude of the systemic inflammatory response after elective operation: a systematic review. Surgery.

[29] Demura S., Takahashi K., Kawahara N., Watanabe Y., Tomita K. (2006). Serum interleukin-6 response after spinal surgery; estimation of surgical magnitude. J Orthop Sci.

[30] Huang T.J., Hsu R.W., Li Y.Y., Chen C.C. (2005). Less systemic cytokine response in patients following microendoscopic versus open lumbar discectomy. J Orthoped Res.

[31] Yang T., Velagapudi R., Terrando N. (2020). Neuroinflammation after surgery: from mechanisms to therapeutic targets. Nat Immunol.

[32] Cheng C., Wan H., Cong P. (2022). Targeting neuroinflammation as a preventative and therapeutic approach for perioperative neurocognitive disorders. J Neuroinflammat.

[33] Gold C., Ray E., Christianson D. (2022). Risk factors for delirium in elderly patients after lumbar spinal fusion. Clin Neurol Neurosurg.

[34] Zhu C., Wang B., Yin J. (2020). Risk factors for postoperative delirium after spinal surgery: a systematic review and meta-analysis. Aging Clin Exp Res.

[35] Zhang H.J., Ma X.H., Ye J.B., Liu C.Z., Zhou Z.Y. (2020). Systematic review and meta-analysis of risk factor for postoperative delirium following spinal surgery. J Orthop Surg Res.

[36] Curtis M., Swan L., Fox R., Warters A., O’Sullivan M. (2023). Associations between body mass index and probable sarcopenia in community-dwelling older adults. Nutrients.

[37] Chang K.V., Hsu T.H., Wu W.T., Huang K.C., Han D.S. (2016). Association between sarcopenia and cognitive impairment: a systematic review and meta-analysis. J Am Med Dir Assoc.

[38] Marzetti E., Picca A., Marini F. (2019). Inflammatory signatures in older persons with physical frailty and sarcopenia: the frailty “cytokinome” at its core. Exp Gerontol.

[39] Deng X., Qin P., Lin Y. (2022). The relationship between body mass index and postoperative delirium. Brain Behav.

[40] Hasemann W., Tolson D., Godwin J., Spirig R., Frei I.A., Kressig R.W. (2018). Nurses’ recognition of hospitalized older patients with delirium and cognitive impairment using the Delirium Observation Screening Scale. A prospective comparison study. J Gerontol Nurs.

[41] Marzetti E., Picca A., Marini F., Biancolillo A., Coelho-Junior H.J., Gervasoni J (2019). Inflammatory signatures in older persons with physical frailty and sarcopenia: The frailty “cytokinome” at its core. Exp Gerontol.

